# Conceptual development and implementation of a digital twin model for managing saltwater intrusion of an island coastal aquifer

**DOI:** 10.1007/s10661-025-14553-x

**Published:** 2025-09-26

**Authors:** Ashneel Sharan, Bithin Datta, Dilip Kumar Roy, Alvin Lal

**Affiliations:** 1 Discipline of Civil Engineering, College of Science & Engineering, James Cook University, Townsville, QLD 4811 Australia; 2 C&R Consulting, Geochemical and Hydrobiological Solutions Pty Ltd., , Townsville, QLD 4814 Australia; 3 Irrigation and Water Management Division, Bangladesh Agricultural Research Institute, Joydebpur, Gazipur 1701 Bangladesh; 4 Global Centre for Environmental Remediation, College of Engineering, Science and Environment, University of Newcastle, Callaghan, NSW 2308 Australia

**Keywords:** Saltwater intrusion, SEAWAT, Ensemble surrogate model, Simulation-optimisation, Digital twin, Pacific Island countries

## Abstract

Saltwater intrusion (SWI) poses a significant environmental challenge for coastal aquifers in Pacific Island nations, including Port Vila, Vanuatu. This study utilised a 3D numerical simulation model to evaluate SWI in the Tagabe coastal aquifer under current pumping regimes. To address SWI, optimal pumping patterns were identified through machine learning-based surrogate ensemble models and a simulation-optimisation (S–O) management model. A digital twin (DT) framework of the Tagabe coastal aquifer was developed, incorporating a 3D numerical model, surrogate ensemble models, and the S–O approach. The DT framework, linked with illustrative field data, was used to generate and analyse five illustrative scenarios based on varying salt concentrations (0.45, 0.55, 0.75, 0.90, and 1.15 kg/m^3^; Scenarios 1 to 5, respectively). The results indicated that scenario 3 (salt concentration of 0.75 kg/m^3^) led to the highest pumping rates from production wells (17,317 m^3^/d) and the lowest from barrier wells (202 m^3^/d), while scenario 5 showed maximum pumping of 31,676 m^3^/d from production wells and 5000 m^3^/d from barrier wells. The S–O model results were validated with less than 10% relative error compared to the numerical model outputs. To the author’s best knowledge, the application of DT in managing SWI has not been applied before. This study is the first to apply a DT framework for managing SWI in coastal aquifers, showcasing its potential for predicting future scenarios and optimising water management strategies. The results from the study indicated that DT can be successfully employed in a coastal aquifer for managing the SWI. The methodology developed and implemented in this study is of global significance and could be used to manage water resources wisely. The study demonstrated that with the help of the S–O approach, the DT is vital in predicting future scenarios, changes in pumping patterns, and other uncertainties.

## Introduction

Saltwater intrusion (SWI) is a major challenge in Pacific Island countries (PICs), and managing SWI is a far more complex and costly process (Sharan et al., 2021). Globally, including PICs, various techniques and methodologies are designed to tackle SWI. However, the severity of the problem keeps on increasing with the changing climate, natural disasters, and human activities. Different strategies for managing SWI in coastal aquifers are needed. However, accessing the field data is becoming costly and time-consuming. Hence, developing the SWI management strategies can get delayed, which would cause the freshwater aquifers to be contaminated with saline water. Therefore, getting the field data about the changes in the hydrogeological parameters and boundary conditions on time is needed. Currently, smart sensors and data loggers feed the data to personal computers (PCs), but they are not yet used in groundwater modelling.

The use of smart sensors in remote monitoring and the feedback information is crucial in managing SWI and water resources. Based on the literature search and the author’s best knowledge, management of SWI using smart techniques has not yet been applied. The current study employs a novel approach to develop and implement a digital twin (DT) model for a coastal aquifer. Based on the literature and the author’s knowledge, the DT model has not been applied globally or in PICs’ SWI management processes. The DT model is emerging as a transformative technology in various industries, offering unparalleled opportunities for optimisation and innovation (Liu et al., 2021). Essentially, a DT is a virtual representation of a physical asset, system, or process that is continuously updated with real-time data (Tao et al., 2022). This dynamic model enables predictive analysis, performance monitoring, and decision support, enhancing operational efficiency and reducing risks. The concept has evolved from its early applications in aerospace to diverse fields such as manufacturing, healthcare, urban planning, and beyond (Attaran & Celik, 2023).

DTs and virtual representations of physical systems have seen growing applications in groundwater management. Ortega (2023) developed a DT model for monitoring the groundwater table in Enschede, Netherlands. The author used smart sensors, which fed the information to the 3D geospatial model through the Internet of Things (IoT). These sensors monitored the groundwater table from rainfall variations, and based on the head heights, the water pumps were activated or deactivated in physical space. The results demonstrated that the DT effectively visualises historical data, current conditions, and anticipated changes in the groundwater table, aiding in identifying areas needing pumping. By integrating forecasts, the system can predict potential groundwater table depths, visualise possible scenarios, and support decision-making processes regarding pumping or retaining groundwater levels through automated messages or physical actions.

Henriksen et al. (2023) developed and implemented the hydrological information and prediction (HIP) system in Denmark. The authors used a combination of real-time data integration, advanced hydrological models, and machine learning techniques to create a DT that can dynamically update and predict hydrological conditions. The study area covered various aspects of Denmark’s water systems, including groundwater levels, soil moisture, and streamflow. The HIP portal, developed by the Danish Agency for Data Supply and Infrastructure, provided a platform for these models and data to be accessed and utilised by various stakeholders, including municipal planners, water supply workers, and environmental consultants. The authors reported that their study highlighted the effectiveness of the HIP DT in improving climate change adaptation, water management, disaster risk reduction, and real-time monitoring and forecasting, helping to identify potential risks and manage water resources more efficiently. The authors recommend further development of the DT approach, emphasising the importance of real-time data updates and collaborative efforts across different sectors to maximise the benefits of this technology for climate resilience and sustainable water management.

Huang et al. (2022) developed a DT of the Earth systems in predicting and analysing floods. The authors used various artificial intelligence (AI), geological, and 3D software to formulate a DT model. Their study focused on surface water hydrology, specifically targeting flood detection and prediction in Earth’s rivers and lakes. The DT framework allows users to explore current Earth system states, predict future conditions, and run hypothetical scenarios to understand potential system evolutions. The authors reported that DT can accurately predict flood events, analyse their impacts, and provide actionable insights for flood management. The authors recommended expanding the DT framework to incorporate more environmental variables and geographic areas and enhancing collaboration between agencies for broader data integration and improved predictive capabilities.

Tsakiridis et al. (2023) developed a DT to assess and monitor soil ecosystems. The authors developed the DT by integrating various data sources, including IoT, sensors, remote sensing data, field measurements, digital cartography, surveys, and other Earth observation data. The integration created a cohesive digital representation of the physical, chemical, and biological characteristics of soil. The authors used multiple environments to test and validate the DT model. The authors reported that the DT could provide detailed insights into soil health, including nutrient cycles, microbial communities, and physical soil structure. The DT framework developed has the potential to do scenario analysis, visualising environmental impacts such as climate change and land use alterations and aiding in decision-making processes for various stakeholders.

Lari et al. (2022) designed a DT to monitor natural source zone depletion (NSZD) of light non-aqueous phase liquids (LNAPLs) at petroleum-impacted sites in Bemidji, Minnesota (USA). The authors used a TMVOCBio (finite volume numerical simulator) multi-phase multi-component multi-microbe transport phenomena at a Darcy scale to mimic the significant NSZD process, hence a DT. The approach integrated partitioning, biodegradation, and fluid dynamics to predict long-term trends and validate the model against field data. The authors reported that the DT accurately simulated complex subsurface processes, providing a detailed and reliable representation of NSZD. The authors also reported that the DT’s ability to predict future depletion trends and support site management decisions underscored its potential utility in their study. The authors recommended further developing and applying DT for complex subsurface systems to enhance decision-making and management strategies for contaminated sites, emphasising the benefits of detailed computational approaches for environmental monitoring and restoration.

The application of DT in groundwater is growing, yet minimal. However, DT has been applied in other disciplines extensively (e.g. Ayani et al., 2018; Uhlemann et al., 2018; Cai et al., 2017; Bottani et al., 2017; Lindstrom et al., 2017; Silva et al., 2023). These studies have shown the exploration and application of the DT paradigm across various domains of manufacturing and production systems. These studies collectively emphasise the integration of real-time data acquisition, sensor data fusion, and cyber-physical systems to create virtual representations, or DTs, of physical entities. This integration enhanced operational efficiency, predictive maintenance, and system optimisation.

SWI in coastal aquifers is a highly dynamic and spatially complex process influenced by sea level rise, groundwater extraction, recharge variability, and aquifer heterogeneity. Traditional numerical models, though powerful, often struggle to provide real-time insights or require frequent recalibration as system conditions change. DT technology, which creates a real-time, continuously updated virtual representation of a physical system, offers a promising new paradigm for managing SWI. By integrating sensor data, simulation models, and predictive analytics, DTs allow for adaptive decision-making, scenario testing, and early warning of potential intrusion events. Despite its growing use in water supply and infrastructure management, the application of DT to SWI remains largely unexplored. This study addresses this gap by demonstrating how a DT framework can enhance monitoring, forecasting, and decision support for managing salinisation risks in vulnerable coastal aquifers.

The application of DT in SWI management in coastal aquifers has not been applied yet. Hence, this paper aims to develop a DT that would be applied to a coastal aquifer of a small Pacific Island country. This study will use a 3D numerical simulation model, various machine learning algorithms, surrogate ensemble models, and optimisation algorithms to design a DT. The detailed steps in the DT model development are given in the section. The DT developed in this study will be used to predict various scenarios for managing the SWI. The advantage of using DT in coastal aquifers is that it can provide real-time feedback from the field, and based on the information, optimal SWI management strategies can be developed. The methodology and findings from this study could aid researchers, policymakers, and water engineers in modelling, planning, and making informed decisions to conserve and utilise the groundwater.

## Methodology

### Study area

The study area for this research is a small Pacific Island country, Vanuatu. Vanuatu, an island nation east of Australia, is comprised of 83 islands with a mix of volcanic mountains and coral atolls. With a population exceeding 300,000, Port Vila’s capital is famous for its natural beauty and attracts tourists. Tourism is crucial to Vanuatu’s economy, contributing 35.5% to its GDP in 2019, alongside agriculture, fishing, and forestry (WTTC, 2021). Despite these economic drivers, Vanuatu faces significant challenges from natural disasters and climate change, leading the government to focus on resilience-building measures (CRCP, 2021; Sharan, 2023; VMGD, 2023).

The six provinces of Vanuatu, including Shefa, where the capital, Efate Island, is located, feature diverse geology influenced by volcanic activity, tectonic movements, and erosion. Efate Island is notable for its volcanic features, such as the Yasur volcano, limestone formations created by marine organisms, and alluvial deposits from nearby rivers (Bath et al., 1986). The island’s topography has been shaped by tectonic movements causing faults and folds and by erosion affecting the low-elevation coastal areas. This geological diversity, coupled with Vanuatu’s susceptibility to natural disasters, highlights the need for integrating climate adaptation and disaster risk reduction into a development plan.

**Fig. 1:**
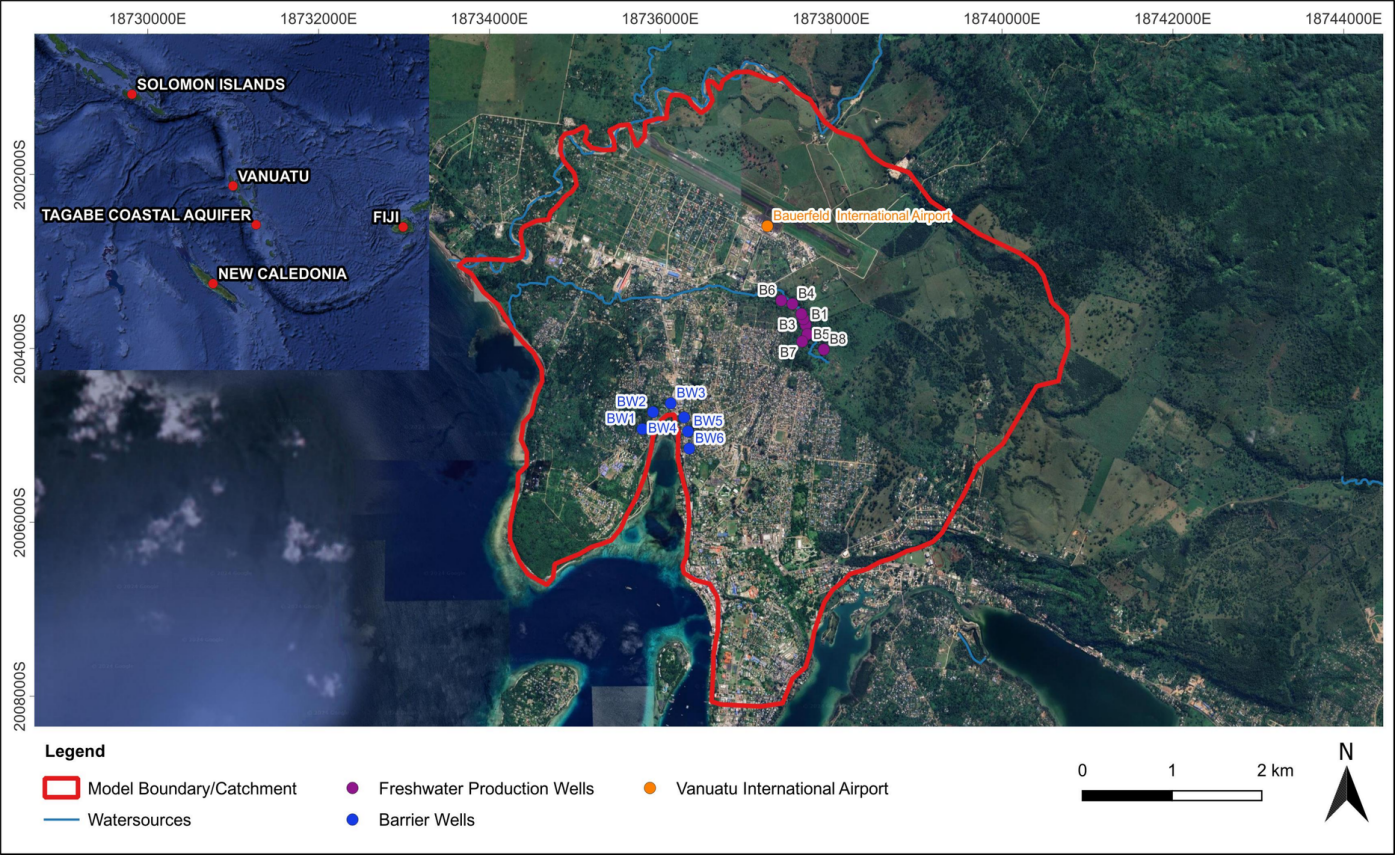
Location of the study area

The methodology developed in this study was applied to the Tagabe coastal aquifer in Vanuatu. The location of the study area is shown in Fig. 1. Eight freshwater wells (B1–B8) pump the freshwater from the Tagabe coastal aquifer, which supplies water in Port Vila. The model boundary or catchment was selected based on the geology and water feeding into the Tagabe coastal aquifer. To minimise the SWI, six barrier wells (BW1-W6) were added near the sea boundary.

### 3D numerical simulation model

Modelling the groundwater processes of a coastal aquifer is a complex and lengthy process because of its heterogeneity. Actuated field data from Tagabe coastal aquifer, including the hydrogeological parameters and boundary conditions, were used to develop a 3D groundwater model. The Tagabe coastal aquifer is a complex, shallow, and unconfined groundwater system influenced by fractured volcanic geology, rainfall, and land use. It supplies water to residents, industries, and agriculture through eight production boreholes. The aquifer consists of two main layers—sand and gravel down to 80 m, underlain by limestone—and is recharged by the Sono and Tagabe rivers. Increasing urbanisation, tourism, and climate change are putting growing pressure on this vital water source, which spans an area of approximately 57 km^2^ (Sharan et al., 2024a).

The groundwater data provided by the Department of Water Resources, Vanuatu, was Limited, consisting of bore pumping data from 2017 to 2022 and electrical conductivity values from 2019 to 2021. Limited data on critical parameters, such as hydraulic head values and initial concentrations, were provided. Despite these Limitations, a 3D numerical simulation model was developed using the available data to manage SWI scenarios in the Tagabe coastal aquifer. The model was calibrated and validated using the salt concentration data (Sharan et al., 2024a). Table 1 details the boundary conditions and hydrogeological parameters used to develop the 3D model.

**Table 1:** Model parameters and the values used for the model design

Definition	Assigned field values	Calibrated values	SI units
Layer 1 (sand and gravel)	Hydraulic conductivity, *K* = 20; porosity = 0.35	Hydraulic conductivity, *K* = 25.5; porosity = 0.45	*K* = m/d; porosity = dimensionless
Layer 2 (limestone)	Hydraulic conductivity, *K* = 35; porosity = 0.45	Hydraulic conductivity, *K* = 40; porosity = 0.55	
Recharge	0.0004	0.000271	m/d
Horizontal anisotropy	1		Dimensionless
Freshwater density	1000		kg/m^3^
Seawater density	1025		kg/m^3^
Saltwater concentration	35		kg/m^3^
Specific storage	0.000035		1/m
River conductance	1		m^2^/dm
Longitudinal dispersivity	30		m
Density/con. constant *F*	0.7143		Dimensionless

Source (Sharan et al., 2024a)

The 3D groundwater model was developed using flow and transport codes in Groundwater Modelling Systems software, explicitly using the SEAWAT code. SEAWAT simulates three-dimensional groundwater flow in porous materials, accommodating variable density and transient conditions by integrating MODFLOW (solves flow model) and MT3DMS (solves solute transport model) using the equations provided by Guo and Langevin (2002).

The conceptual model of the Tagabe coastal aquifer was created with MODFLOW, covering a model domain of 7.50 km × 7.61 km × 0.1 km. A 3D finite-difference grid with 100 × 100 m cells was used, consisting of two layers with different hydraulic conductivities and porosities. Layer 1 (0 to −80 m) had a hydraulic conductivity of 20 m/d and a porosity of 0.35, while layer 2 (−80 to −100 m) had a hydraulic conductivity of 35 m/d and a porosity of 0.45. Boundary conditions included a specified head boundary (CHB) on the southern side, a no-flow boundary on the eastern side, and the Sono River on the northwestern boundary connected to the ocean boundary.

Due to the lack of observed hydraulic head values from boreholes in Tagabe, approximate values were assigned based on available data. CHB values for the western and southeastern nodes were set at 9 m and 8.8 m, respectively, maintaining a slight hydraulic gradient towards the sea. Head values for the Sono River and Tagabe River nodes were assigned accordingly, considering the presence of salinity in the aquifer. Boreholes B1–B8 (Fig. 1) were included in the model domain, with transient pumping values from 2017 to 2021. All hydrogeological parameters, boundary conditions, wells, pumping values, and head values were integrated into MODFLOW, and a 20-year steady-state simulation was conducted to establish initial conditions.

SEAWAT was initiated for a 5-year period, utilising pumping data from 2017 to 2021. The model was calibrated using electrical conductivity (EC) data from boreholes B1, B2, B3, B7, and B8 from 2019 to 2020 (2021 data missing). Despite the lack of observed head values, calibration was performed using secondary data through trial-and-error methods, adjusting initial salt concentrations, recharge values, hydraulic conductivities, and porosities (Sharan et al., 2024a). After calibration and validation, the model was simulated for the next 20 years (2022–2043), with the calibrated results serving as the base case for the study. The sensitivity analysis was also carried out by changing the hydraulic conductivities and simulating the model (Sharan et al., 2024a). The calibrated model was also used to obtain output (salt concentrations) values at four monitoring locations (MLs) using randomly generated input (pumping) values. The four MLs were selected from the current production wells. Hence, ML1 = B1, ML2 = B3, ML3 = B7, and ML4 = B8 (Fig. 1).

The 3D numerical model was run 130 times using different spatiotemporal pumping patterns to determine the salt concentration at four monitoring sites. These pumping patterns served as the inputs, while the resulting salt concentrations were the outputs. Using these input–output patterns, this study developed an ensemble of several machine learning-based surrogate models. The limited number of input–output patterns aimed to reduce computational demands in the coupled S–O approach and to evaluate whether a small dataset, despite having 70 variables, could effectively and accurately train the surrogate ensemble models.

### Surrogate ensemble model

Machine learning-based surrogate and ensemble models were developed to replicate the 3D numerical simulation model of the Tagabe coastal aquifer. Various machine learning and deep learning algorithms can be employed to build these surrogate and ensemble models (Asher et al., 2015; Song et al., 2011). This study developed an ensemble of several surrogate models as approximations of the numerical simulation model. Notably, this study pioneers the use of limited datasets to train multiple machine learning models as surrogate ensembles and the application of decision theories to choose the best surrogate ensemble model.

The machine learning and deep learning algorithms used to develop surrogate ensemble models were Artificial Neural Network (ANN), Support Vector Regression (SVR), Genetic Programming (GP), MARS_C = Multivariate Adaptive Regression Spline with Piecewise Cubic Spline, MARS_L = Multivariate Adaptive Regression Spline with Piecewise Linear Spline, Probabilistic Linear Regression (PLR), Random Forest Regression (RFR), Group Method of Data Handling (GMDH), Long Short-Term Memory (LSTM), Adaptive Neuro-Fuzzy Inference Systems (ANFIS), Asynchronous Successive Halving Algorithm Tuned Automatic Model Selection (AMS-AHSA), and Bayesian Optimisation Tuned Automatic Model Selection (AMS-Bayesian).

The input–output patterns generated from the 3D numerical simulation model were used to train the machine learning and deep learning models mentioned above.

### Model performance

Several performance evaluation criteria were employed to assess the surrogate models. These included the correlation coefficient or Pearson’s correlation coefficient (*R*^2^) (PCC), Index of Agreement (IoA), Kling-Gupta efficiency (KGE), Nash–Sutcliffe efficiency coefficient (NSE), mean absolute error (MAE), root mean square error (RMSE), and mean absolute percentage error (MAPE). These metrics were used to evaluate both surrogate and ensemble models.

Moreover, Shannon’s entropy-based decision theory was used to select the best-performing model (Shannon, 1948). Using the steps outlined in Li et al. (2011), the model ranking was done. After ranking the best surrogate models, the first five ranked surrogates were used to form an ensemble model for each monitoring location.

### Management model

**Fig. 2:**
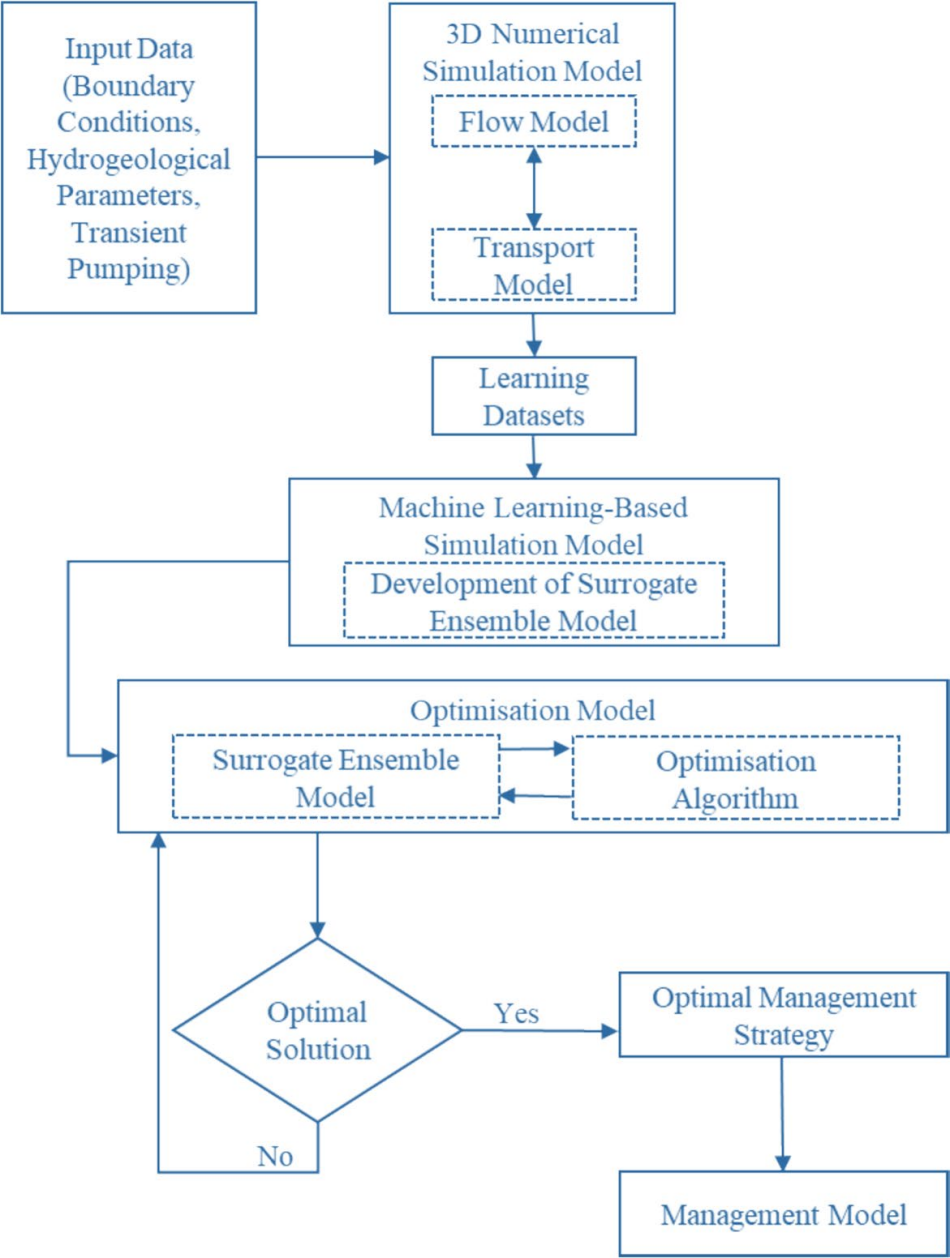
Flow diagram of linked simulation-optimisation management model

Using the ensemble models developed in the “Surrogate ensemble model” section, a management model or simulation-optimisation (S–O) model was developed. The ensemble models were externally linked with the Controlled Elitist Multiple Objective Genetic Algorithm (CEMOGA) (Deb & Goel, 2001) to find the optimal groundwater management pumping values. The flow diagram of developing a management model using 3D numerical simulation and surrogate ensemble models is given in Fig. 2.

The management model developed in this study addresses a multi-objective problem with two conflicting objectives. The objectives are to maximise freshwater extraction from PWs for beneficial use and to minimise pumping from BWs. Reducing BW pumping is important because disposing of large volumes of saline water can be problematic. According to Datta and Peralta (1986), the solutions from the management model will yield a Pareto optimal front, offering water managers and policymakers a clear trade-off between the two conflicting objectives. The mathematical representation of this management model is provided in Eqs. (1) to (6) (Bhattacharjya & Datta, 2005).

1 $$\textrm{Maximise}:\kern0.5em {f}_1\left({Q}_{PW}\right)={\sum}_{n=1}^N{\sum}_{t=1}^T{Q}_{PW_n}^t$$

2 $$\textrm{Minimise}\kern0.5em {f}_2\left({Q}_{BW}\right)=\sum_{m=1}^M\sum_{t=1}^T{Q}_{BW_m}^t$$

Subject to

3 $${C}_i=\varepsilon \left({Q}_{PW},{Q}_{BW}\right)$$

4 $${C}_i\le {C}_{max}{\forall}_i$$

5 $${Q}_{PW_{min}}\le {Q}_{PW_n}^t\le {Q}_{PW_{max}}$$

6 $${Q}_{BW_{min}}\le {Q}_{BW_n}^t\le {Q}_{BW_{max}}$$

where PW = production well, BW = barrier well; where ${Q}_{{\text{PW}}_{\text{n}}}^{\text{t}}$ = water extraction from the *n*th production well during *t*th time steps; ${Q}_{{\text{BW}}_{\text{m}}}^{\text{t}}$ = water extraction from the *n*th barrier well during *t*th time steps; ${C}_{\text{i}}$ = saltwater concentrations at the *i*th MLs at the end of the management period; $\varepsilon ()$ denotes the density-dependent coupled flow and salt transport surrogate model. The constraint given in Eq. 3 shows the linking of the surrogate ensemble model with the optimisation model; the constraint given in Eq. 4 indicates the maximum allowable salt concentration at various MLs; the constraints given in Eqs. 5 and 6 outline the upper and lower limits of the water abstraction from pumping and barrier wells, respectively.

### Digital twin

**Fig. 3:**
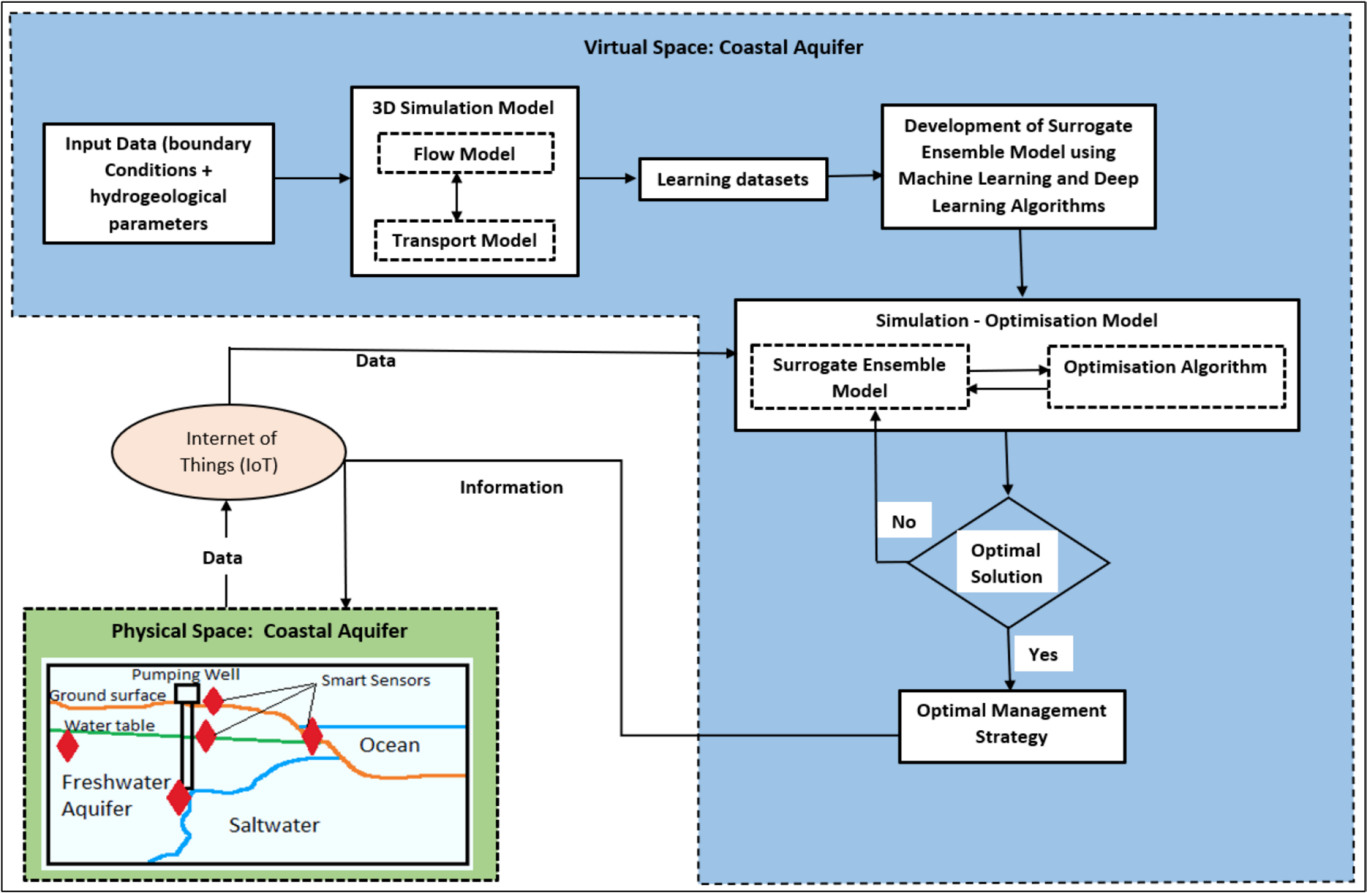
Digital twin framework of a coastal aquifer

In this study, the virtual representation of a coastal aquifer was used using the digital twin (DT) framework. The DT framework and model design are shown in Fig. 3. The development of the DT for a coastal aquifer is a complex and challenging task. Initially, the 3D numerical simulation model was developed using the field data as described in the “3D numerical simulation model” section. Secondly, using the learning datasets, 12 surrogate and four ensemble models were developed (the “Surrogate ensemble model” section). Thirdly, a management model (S–O model) was developed using the ensemble model, MOGA optimisation algorithm, and sets of constraints (Eqs. 1 to 6) (the “Management model” section). These three models represent the virtual space component of the DT framework. The physical space of the DT framework represents the actual coastal aquifer (Tagabe coastal aquifer).

The DT is where the physical and virtual spaces are connected. Hence, the smart sensors supply the field data through the Internet of Things (IoTs) to the computers where the virtual model is located. Using the field data supplied by the IoTs, new sets of constraints (Eqs. 1 to 6) are set, and the optimal management strategy is developed for managing the saltwater intrusion. Due to time, budget, and government approval constraints, the smart sensors were not placed in Tagabe coastal aquifer. Instead, five illustrative sensor scenarios were developed using salt concentration thresholds of 0.45, 0.55, 0.75, 0.90, and 1.15 kg/m^3^. These values were chosen to simulate increasing severity of saltwater intrusion, ranging from initial detection to high salinity levels. The thresholds were informed by (i) observed salinity ranges in the region based on historical groundwater data, (ii) conversion of chloride concentration benchmarks from WHO and local guidelines, and (iii) published values in similar coastal aquifer studies. This approach allowed the DT to be tested under realistic conditions despite the absence of field-deployed smart sensors. It was assumed that the smart sensors were placed in the field to measure the standing water levels in bores, rainfall (recharge) rates, and the salt concentration of the water, as illustrated in Fig. 3. These sensors fed the data daily to the computer hardware, and based on this data, a new set of optimal groundwater pumping rates was decided at the end of every month. The governing body implemented the optimal pumping rates to the groundwater users in Port Vila.

The physical space (actual aquifer) has smart sensors, constantly monitoring the water levels, salinity, and rainfall rates in Tagabe coastal aquifer. These field data are transmitted using IoT to the surrogate ensemble model, which then re-runs and gives different sets of Pareto optimal fronts (optimal pumping values). The optimal management strategy is then fed back to the physical space. For instance, the smart sensors recorded the drop in groundwater level. Hence, the constraints have to be changed, and the maximum pumping limit has to be reduced in Eqs. 1 and 2 due to the drop in the groundwater level. Using modified constraints, the optimisation algorithm will provide the optimal pumping values, which will then be passed to the users of groundwater. These optimal pumping values would be safe and would prevent saltwater intrusion in coastal aquifers. For this study, the DT works in the steps discussed above.

Using the physical space data provided by IoTs and the constraints outlined in Eqs. 1 to 6, five scenarios were simulated using the DT for Tagabe coastal aquifer. The sensors have triggered and sent five sets of data/scenarios. The sensors that were illustratively placed in the field measured the salt concentrations of the groundwater in four different MLs and sent the data to the computer, which has the virtual system in place. Based on the new maximum allowable concentrations, Pareto optimal pumping values were simulated. The five scenarios/trigger limits given by the field sensors include the following:

- *Scenario 1:* The maximum allowable salt concentration has decreased from 0.5 to 0.45 kg/m^3^. The concentration, 0.5 kg/m^3^, was estimated from the numerical model. Based on this new constraint, the total allowable salt concentration is 0.45 kg/m^3^. However, the maximum (1200 m^3^/d) and minimum pumping (0 m^3^/d) remain the same. The 12 surrogates and four ensemble models developed in the “Surrogate ensemble model” section were used to optimise the pumping rate for this scenario. However, the Pareto optimal fronts were generated using the 0.45 kg/m^3^ allowable salt concentration at all MLs.
- *Scenario 2:* The maximum allowable salt concentration has increased from 0.45 to 0.55 kg/m^3^. Based on this new constraint, the total allowable salt concentration is 0.55 kg/m^3^. However, the maximum (1200 m^3^/d) and minimum pumping (0 m^3^/d) remain the same. The surrogate models used in scenario 1 were used to optimise the pumping rate for this scenario. However, the Pareto optimal fronts were generated using the 0.55 kg/m^3^ allowable salt concentration at all MLs.
- *Scenario 3:* The maximum allowable salt concentration has increased from 0.55 to 0.75 kg/m^3^. Based on this new constraint, the total allowable salt concentration is 0.75 kg/m^3^. However, the maximum (1200 m^3^/d) and minimum pumping (0 m^3^/d) remain the same. The surrogate models used in scenario 1 were used to optimise the pumping rate for this scenario. However, the Pareto optimal fronts were generated using the 0.75 kg/m^3^ allowable salt concentration at all MLs.
- *Scenario 4:* The maximum allowable salt concentration has increased from 0.75 to 0.9 kg/m^3^. Based on this new constraint, the total allowable salt concentration is 0.9 kg/m^3^. However, the maximum (1200 m^3^/d) and minimum pumping (0 m^3^/d) remain the same. The surrogate models used in scenario 1 were used to optimise the pumping rate for this scenario. However, the Pareto optimal fronts were generated using the 0.9 kg/m^3^ allowable salt concentration at all MLs. 
- *Scenario 5:* The maximum allowable salt concentration has increased from 0.9 to 1.1 kg/m^3^. Based on this new constraint, the total allowable salt concentration is 1.1 kg/m^3^. However, the maximum (1200 m^3^/d) and minimum pumping (0 m^3^/d) remain the same. The surrogate models used in scenario 1 were used to optimise the pumping rate for this scenario. However, the Pareto optimal fronts were generated using the 1.1 kg/m^3^ allowable salt concentration at all MLs.

These scenarios were simulated using four different surrogate ensembles and the S–O model for different constraints. The Pareto optimal front values were generated, and with the help of the local government, water managers, and local municipal office, these optimal pumping rates could be sent to the groundwater users of Tagabe coastal aquifer. The optimal pumping rates would be sent to the users using the smart information boards placed near the nested bore networks by IoTs. These smart information boards would show the optimal pumping rates based on different scenarios. The use of machine learning, smart sensors, DT, and smart information boards will significantly reduce remote travel, costs, and time and improve the sustainability of groundwater resources in Tagabe coastal aquifers and other aquifers as well.

## Results and discussion

### 3D numerical simulation model

**Fig. 4:**
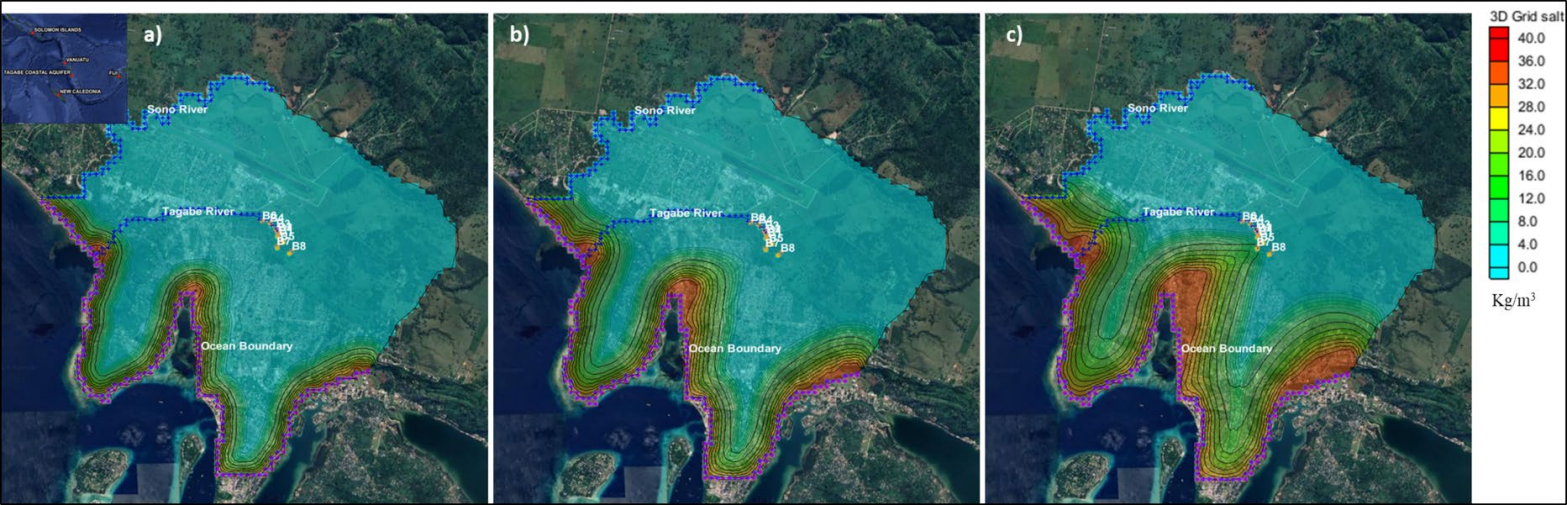
Numerical model simulated saltwater intrusion rates in Tagabe coastal aquifer with production bores (B1-B8): **a** length of saltwater intrusion after 5 years of current pumping regimes in Tagabe; **b** length of saltwater intrusion after 10 years of current pumping regimes in Tagabe; **c** length of saltwater intrusion after 20 years of current pumping regimes in Tagabe

**Fig. 5:**
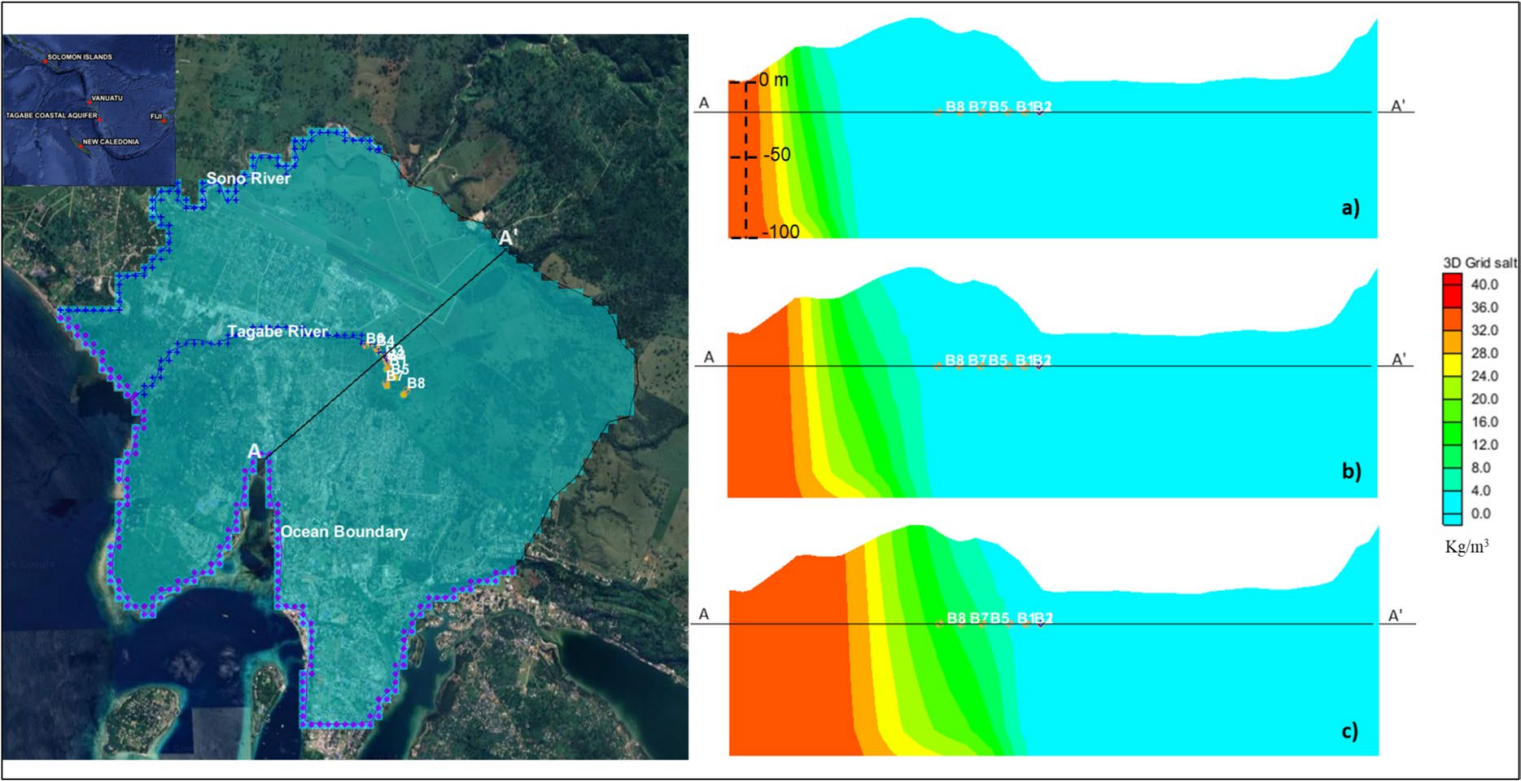
Side view cross-sections of salt concentration of layer 1 with production bores (B1–B8): **a** length of saltwater intrusion after 5 years of current pumping rates in Tagabe; **b** length of saltwater intrusion after 10 years of current pumping rates in Tagabe; **c** length of saltwater intrusion after 20 years of current pumping rates in Tagabe

The numerical simulation model was developed, calibrated, and validated using the salt concentration data. Using the calibrated data and the current pumping regimes, the numerical model was simulated for 20 years, starting from 2022 to 2043. The extent of SWI for three time steps (5, 10, and 20 years) is shown in Fig. 4. The length of SWI is clearly shown in the figure. With the current pumping regime, the wells would be contaminated, and the freshwater Tagabe aquifer would have saline water with concentrations reaching up to 20 kg/m^3^ (20,000 mg/L). Moreover, Fig. 5 shows the cross-sections of the SWI extents/lengths. These cross-sections clearly show when the saline water would reach the production wells (B1–B8). Hence, the water would not be suitable for drinking and irrigation.

Therefore, managing SWI is essential as Tagabe coastal aquifer supplies fresh water to the capital city (Port Vila). The use of barrier wells, injection/recharge wells, and managing pumping rates was effective for managing SWI in Tagabe coastal aquifer (Sharan et al., 2024a). However, knowing the optimum pumping rates from barrier wells (BWs) and production wells (PWs) is challenging and cannot be fully achieved by the numerical simulation model. Also, optimising the numerical simulation model would take an immense amount of computational time (Bhattacharjya & Datta, 2005). Hence, this study developed a methodology to maximise the pumping from the PW and minimise the pumping from the BW, maintaining the maximum allowable salt concentration. The machine learning surrogate ensembles and S–O models were developed to tackle the multi-objective simulation-optimisation problem. However, the main aim of this study is to develop a digital twin (DT) of Tagabe coastal aquifer. Hence, machine learning surrogate ensembles and S–O models need to be developed initially to develop the DT.

### Machine learning surrogate ensemble model

**Fig. 6:**
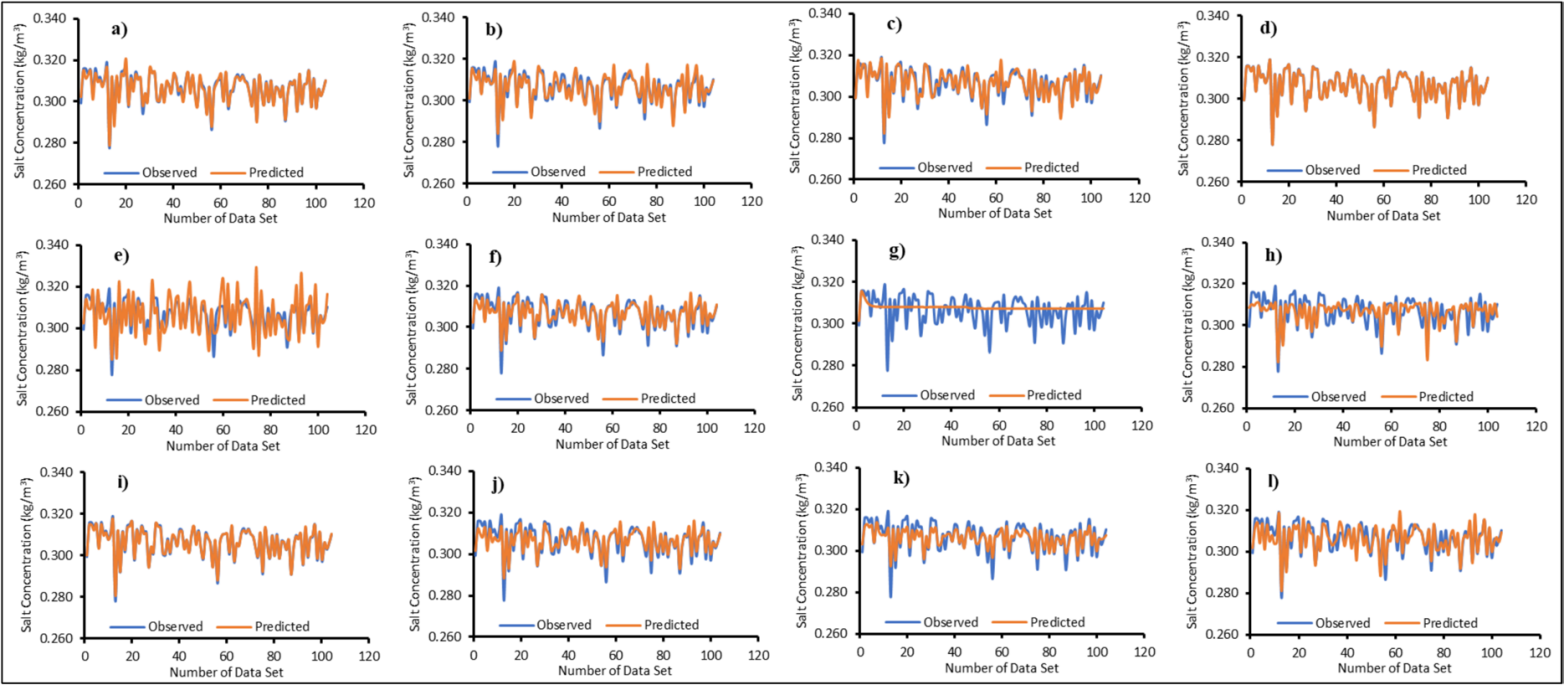
Actual versus predicted salt concentrations of monitoring location 1 (ML1) using different machine learning algorithms during the training period; **a**—Adaptive Neuro Fuzzy Inference System (ANFIS), **b**—Artificial Neural Network (ANN), **c**—Multivariate Adaptive Regression Spline with Piecewise Cubic Spline (MARS_C), **d**—Multivariate Adaptive Regression Spline with Piecewise Linear Spline (MARS_L), **e**—Bayesian Optimization tuned Automatic Model Selection (AMS_Bayesian), **f**—Asynchronous Successive Halving Algorithm tuned Automatic Model Selection (AMS_ASHA), **g**—Bi-directional Long Short-Term Memory Network (Bi-LSTM), **h**—Group Method of Data Handling (GMDH), **i**—Gaussian Process Regression (GPR), **j**—Probabilistic Linear Regression (PLR), **k**—Random Forest (RF) and **l**—Support Vector Regression (SVR)

**Fig. 7:**
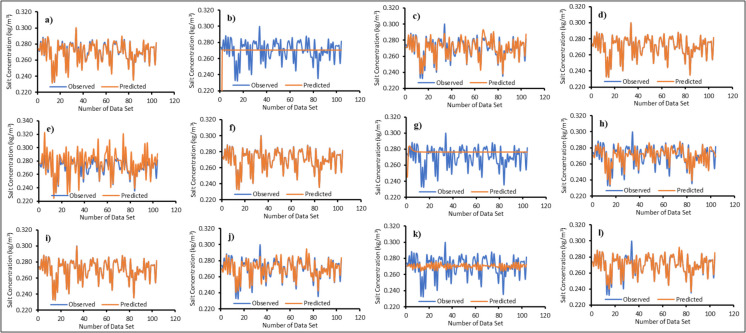
Actual versus predicted salt concentrations of monitoring location 2 (ML2) using different machine learning algorithms during the training period; **a**—Adaptive Neuro Fuzzy Inference System (ANFIS), **b**—Artificial Neural Network (ANN), **c**—Multivariate Adaptive Regression Spline with Piecewise Cubic Spline (MARS_C), **d**—Multivariate Adaptive Regression Spline with Piecewise Linear Spline (MARS_L), **e**—Bayesian Optimization tuned Automatic Model Selection (AMS_Bayesian), **f**—Asynchronous Successive Halving Algorithm tuned Automatic Model Selection (AMS_ASHA), **g**—Bi-directional Long Short-Term Memory Network (Bi-LSTM), **h**—Group Method of Data Handling (GMDH), **i**—Gaussian Process Regression (GPR), **j**—Probabilistic Linear Regression (PLR), **k**—Random Forest (RF) and **l**—Support Vector Regression (SVR)

**Fig. 8:**
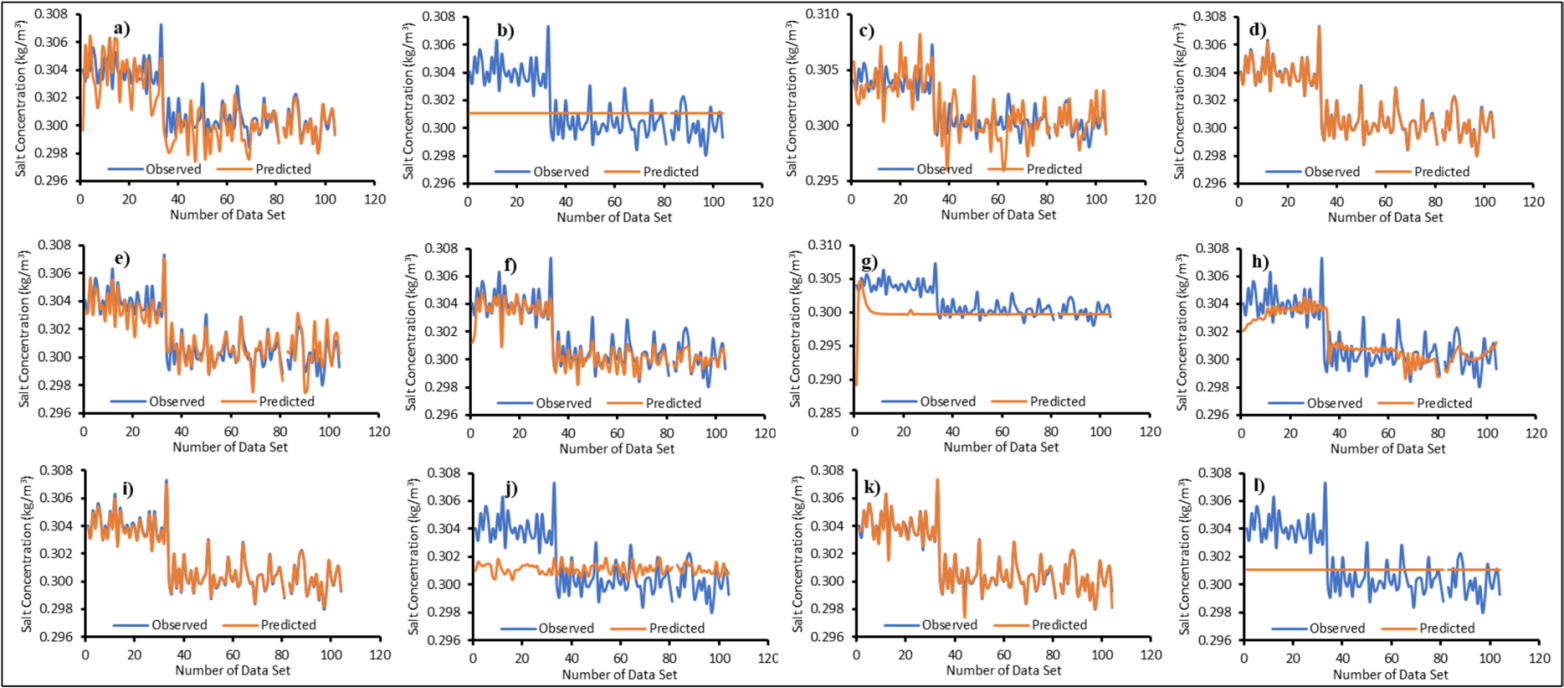
Actual versus predicted salt concentrations of monitoring location 3 (ML3) using different machine learning algorithms during the training period; **a**—Adaptive Neuro Fuzzy Inference System (ANFIS), **b**—Artificial Neural Network (ANN), **c**—Multivariate Adaptive Regression Spline with Piecewise Cubic Spline (MARS_C), **d**—Multivariate Adaptive Regression Spline with Piecewise Linear Spline (MARS_L), **e**—Bayesian Optimization tuned Automatic Model Selection (AMS_Bayesian), **f**—Asynchronous Successive Halving Algorithm tuned Automatic Model Selection (AMS_ASHA), **g**—Bi-directional Long Short Term Memory Network (Bi-LSTM), **h**—Group Method of Data Handling (GMDH), **i**—Gaussian Process Regression (GPR), **j**—Probabilistic Linear Regression (PLR), **k**—Random Forest (RF) and **l**—Support Vector Regression (SVR)

**Fig. 9:**
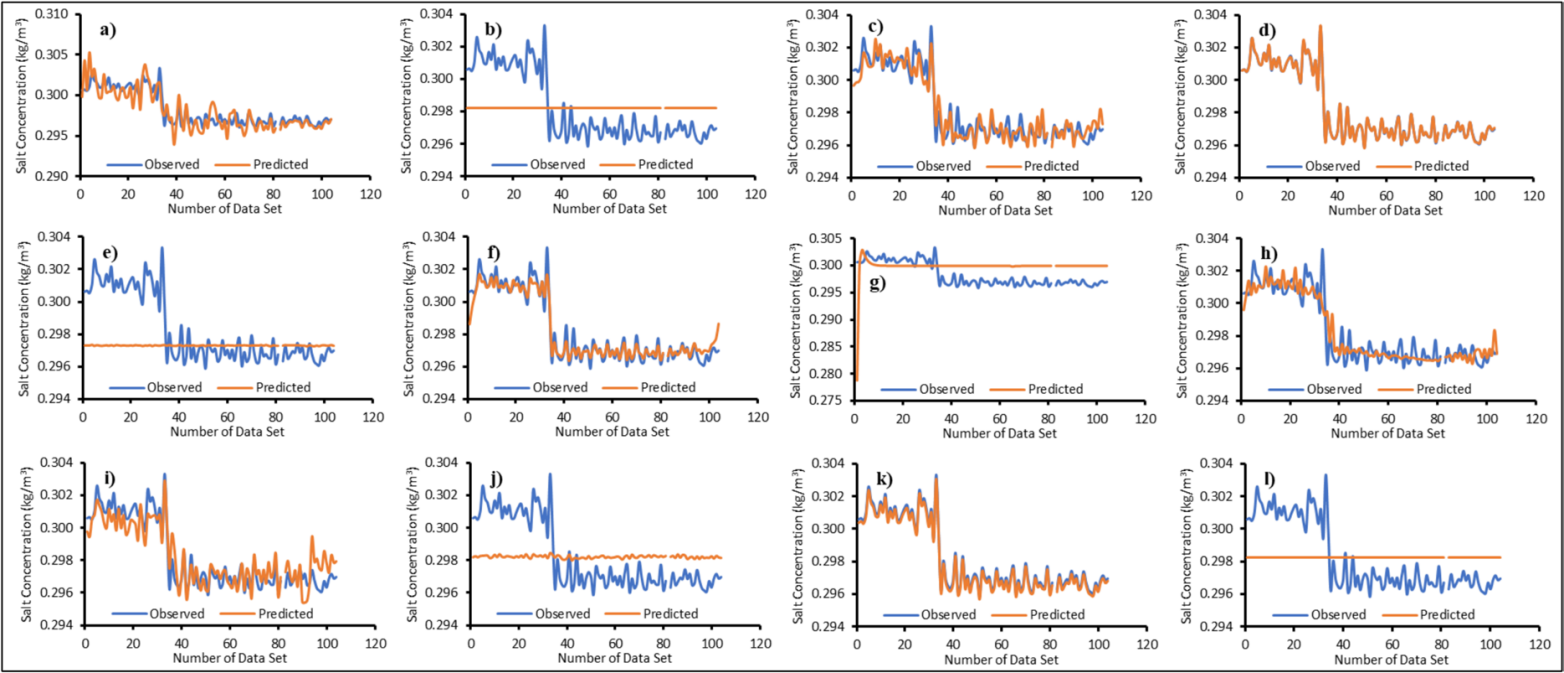
Actual versus predicted salt concentrations of monitoring location 4 (ML4) using different machine learning algorithms during the training period; **a**—Adaptive Neuro Fuzzy Inference System (ANFIS), **b**—Artificial Neural Network (ANN), **c**—Multivariate Adaptive Regression Spline with Piecewise Cubic Spline (MARS_C), **d**—Multivariate Adaptive Regression Spline with Piecewise Linear Spline (MARS_L), **e**—Bayesian Optimization tuned Automatic Model Selection (AMS_Bayesian), **f**—Asynchronous Successive Halving Algorithm tuned Automatic Model Selection (AMS_ASHA), **g**—Bi-directional Long Short-Term Memory Network (Bi-LSTM), **h**—Group Method of Data Handling (GMDH), **i**—Gaussian Process Regression (GPR), **j**—Probabilistic Linear Regression (PLR), **k**—Random Forest (RF) and **l**—Support Vector Regression (SVR)

Twelve surrogate models were trained using the machine learning algorithms mentioned in the “Surrogate ensemble model” section. Figures 6, 7, 8, and 9 illustrate the actual versus predicted results during the training phase. A limited dataset was chosen to enhance computational efficiency when running the numerical model. One of the innovative aspects of this study is the use of a limited number of input–output patterns. The results demonstrated that even with limited training data, the surrogates were able to predict salt concentrations with reasonable accuracy for all MLs, as shown in Figs. 6, 7, 8, and 9. The majority of the surrogate models performed exceptionally well during the model training stage, with few giving poor prediction results. The following surrogates did not perform well in the training stage: BiLSTM for ML1; ANN, BiLSTM, and RF for ML2; ANN, BiLSTM, PLR, and SVR for ML3; ANN, AMS_Bayesian, BiLSTM, PLR, and SVR for ML4. These surrogates did not do well in predicting the salt concentrations at various MLs. However, to further investigate and find the accuracy in model prediction, different performance measures were employed, as outlined in the “Model performance” section.

### Model performance

**Fig. 10:**
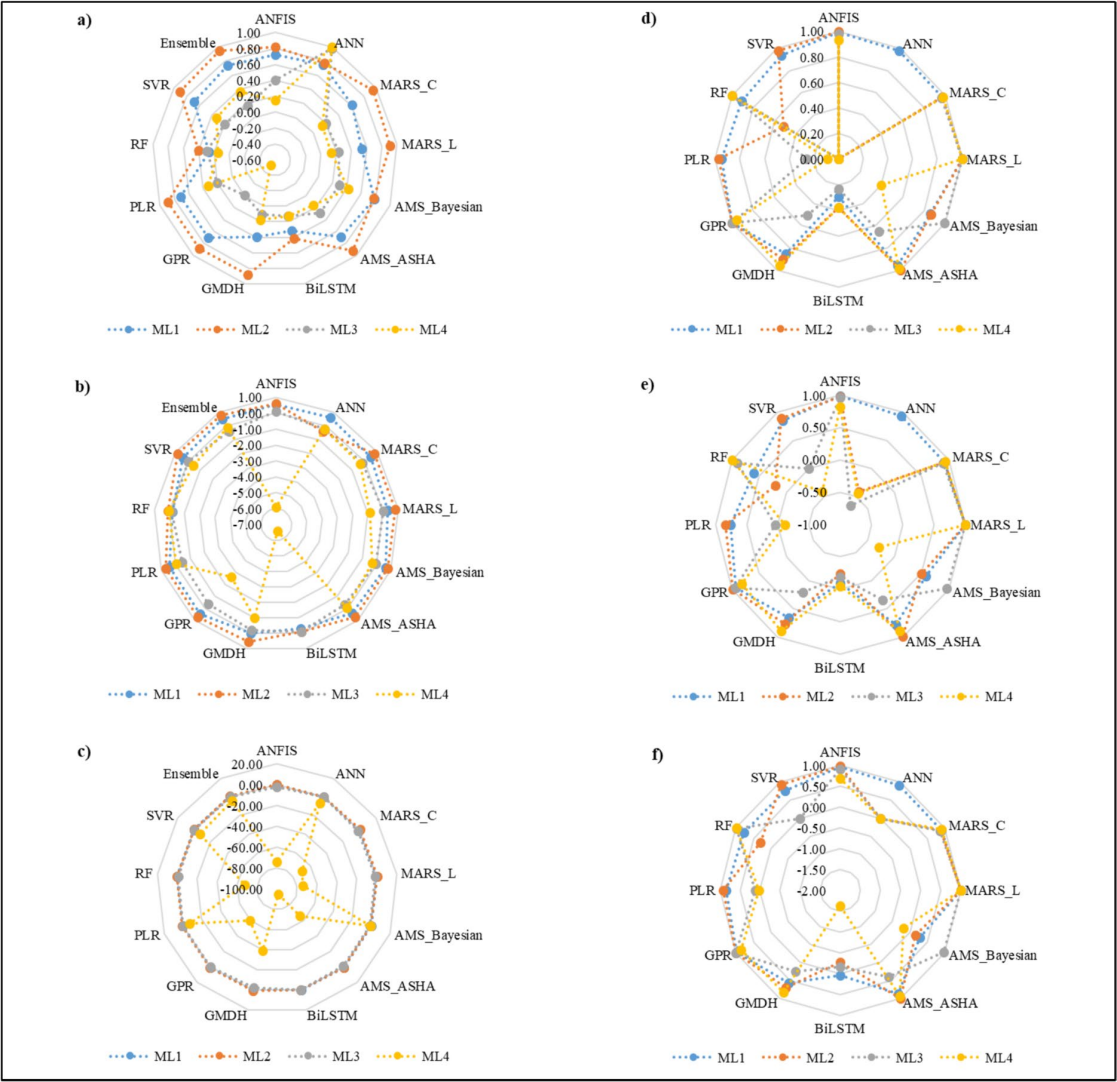
Spider plots of three major performance measures of 12 surrogate models for different monitoring locations: **a** Index of Agreement values for 12 surrogate models during the testing period; **b** Kling-Gupta Efficiency Coefficient values for 12 surrogate models during the testing period; **c** Nash Sutcliffe Efficiency Coefficient values for 12 surrogate models during the testing period; **d** Index of Agreement values for 12 surrogate models during the training period; **e** Kling-Gupta Efficiency Coefficient values for 12 surrogate models during the training period; **f** Nash Sutcliffe Efficiency Coefficient values for 12 surrogate models during the training period

The criteria used to assess the performance of the surrogate models for various monitoring locations during the training stage are detailed in Table 2 and Fig. 10d–f. These performance metrics focus on the training phase, where the predicted salt concentration values from the different ML models described in the “3D numerical simulation model” section were compared with the actual measurements. Similar techniques were used, and results were reported by Roy et al. (2020) and Roy et al. (2024). Most models showed improved accuracy in predicting salt concentrations during the training phase. When examining the mean absolute percentage error (MAPE), Pearson’s coefficient, Index of Agreement, Kling-Gupta efficiency, and Nash Sutcliffe efficiency, it is evident that ANFIS, ANN, MARS_C, MARS_L, AMS_ASHA, GMDH, RF, GPR, and SVR excelled in predicting salt concentrations at ML1 during the training stage. Similarly, ANFIS, MARS_C, MARS_L, AMS_ASHA, GPR, PLR, and SVR were more effective at predicting salt concentrations at ML2. At ML3, ANFIS, MARS_C, MARS_L, AMS_Bayesian, GPR, and RF outperformed others in the training stage predictions. Lastly, at ML4, MARS_C, MARS_L, AMS_ASHA, GMDH, and RF demonstrated superior performance during training in predicting salt concentrations.

**Table 2:** Performance criteria of 12 surrogate models at four different monitoring locations (MLs) during the model training period

	**Performance measure**	**ANFIS**	**ANN**	**MARS_C**	**MARS_L**	**AMS_Bayesian**	**AMS_ASHA**	**BiLSTM**	**GMDH**	**GPR**	**PLR**	**RF**	**SVR**
**ML1**	Mean Absolute Deviation (MAD)	0.0009293	0.0017723	0.0017369	0.0000927	0.0048193	0.0020226	0.0052974	0.0038625	0.0005386	0.0021990	0.0025491	0.0028542
	Mean Square Error (MSE)	0.0000017	0.0000045	0.0000052	0.0000000	0.0000373	0.0000067	0.0000495	0.0000223	0.0000005	0.0000078	0.0000117	0.0000118
	RMSE	0.0012968	0.0021187	0.0022825	0.0001138	0.0061111	0.0025953	0.0070324	0.0047200	0.0006848	0.0027999	0.0034223	0.0034335
	MAPE (%)	0.303	0.580	0.568	0.030	1.570	0.663	1.754	1.263	0.176	0.720	0.837	0.932
**ML2**	Mean Absolute Deviation (MAD)	0.001235	0.010136	0.003231	0.000171	0.009189	0.000074	0.010648	0.005414	0.000100	0.002983	0.008319	0.001147
	Mean Square Error (MSE)	0.000003	0.000168	0.000017	0.000000	0.000141	0.000000	0.000214	0.000050	0.000000	0.000015	0.000117	0.000011
	RMSE	0.001600	0.012966	0.004155	0.000212	0.011885	0.000101	0.014639	0.007071	0.000128	0.003913	0.010814	0.003376
	MAPE (%)	0.459	3.840	1.197	0.063	3.371	0.027	4.102	2.020	0.037	1.114	3.142	0.433
**ML3**	Mean Absolute Deviation (MAD)	0.0009339	0.0020962	0.0012951	0.0000495	0.0005557	0.0008921	0.0023394	0.0013296	0.0001112	0.0020018	0.0001535	0.0020977
	Mean Square Error (MSE)	0.0000017	0.0000198	0.0000028	0.0000000	0.0000005	0.0000121	0.0000230	0.0000150	0.0000001	0.0000173	0.0000003	0.0000198
	RMSE	0.0012984	0.0044534	0.0016758	0.0000625	0.0007043	0.0034716	0.0047910	0.0038725	0.0002292	0.0041651	0.0005843	0.0044534
	MAPE (%)	0.309	0.714	0.433	0.016	0.185	0.313	0.791	0.459	0.038	0.681	0.054	0.714
**ML4**	Mean Absolute Deviation (MAD)	0.0008730	0.0018996	0.0004841	0.0000299	0.0016707	0.0003444	0.0026600	0.0006084	0.0006741	0.0018773	0.0001878	0.0019016
	Mean Square Error (MSE)	0.0000013	0.0000045	0.0000004	0.0000000	0.0000053	0.0000002	0.0000117	0.0000007	0.0000007	0.0000043	0.0000000	0.0000045
	RMSE	0.0011613	0.0021146	0.0006191	0.0000366	0.0023100	0.0004946	0.0034221	0.0008436	0.0008286	0.0020803	0.0001883	0.0021146
	MAPE (%)	0.292	0.636	0.162	0.010	0.557	0.115	0.893	0.204	0.226	0.628	0.063	0.636

Source (Sharan et al., 2024b)

Table 3 and Fig. 10a–c highlight the performance of 12 surrogate models across various MLs during the testing phase. The actual and predicted salt concentrations were compared using metrics such as MAPE (%), Pearson’s correlation coefficient, Index of Agreement, Kling-Gupta efficiency, and Nash–Sutcliffe efficiency. At ML1, the models ANN, AMS_Bayesian, and PLR demonstrated superior predictive accuracy. For ML2, the models ANFIS, MARS_C, MARS_L, AMS_Bayesian, AMS_ASHA, GHD, and SVR consistently produced more accurate predictions. At ML3, AMS_Bayesian and ANN were the most reliable, with a strong performance from ANFIS, AMS_ASHA, and SVR.

**Table 3:** Performance criteria of 12 surrogate models and four ensemble models at four different monitoring locations (MLs) during the model testing period

	**Performance measure**	**ANFIS**	**ANN**	**MARS_C**	**MARS_L**	**AMS_Bayesian**	**AMS_ASHA**	**BiLSTM**	**GMDH**	**GPR**	**PLR**	**RF**	**SVR**	**Ensemble**
**ML1**	Mean Absolute Deviation (MAD)	0.008200	0.006349	0.007465	0.008784	0.006097	0.005403	0.006612	0.006838	0.005481	0.005194	0.006468	0.005891	0.005640
	Mean Square Error (MSE)	0.000106	0.000072	0.000111	0.000149	0.000060	0.000068	0.000121	0.000112	0.000066	0.000062	0.000106	0.000074	0.000064
	RMSE	0.010318	0.008478	0.010540	0.012212	0.007752	0.008220	0.011006	0.010560	0.008094	0.007874	0.010290	0.008610	0.007997
	MAPE (%)	2.718	2.072	2.528	2.965	2.045	1.839	2.270	2.333	1.861	1.767	2.210	1.998	1.909
**ML2**	Mean Absolute Deviation (MAD)	0.01127	0.01195	0.00480	0.00693	0.01124	0.00513	0.01089	0.00620	0.00629	0.00611	0.01070	0.00590	0.00383
	Mean Square Error (MSE)	0.00016	0.00020	0.00003	0.00007	0.00019	0.00005	0.00026	0.00006	0.00007	0.00006	0.00016	0.00006	0.00003
	RMSE	0.01282	0.01408	0.00570	0.00819	0.01364	0.00682	0.01603	0.00770	0.00815	0.00783	0.01251	0.00767	0.00548
	MAPE (%)	4.169	4.465	1.808	2.569	4.232	1.945	4.245	2.345	2.396	2.330	4.036	2.237	1.469
**ML3**	Mean Absolute Deviation (MAD)	0.006016	0.001744	0.004818	0.005643	0.003692	0.003950	0.002270	0.005475	0.002075	0.001717	0.003989	0.001750	0.001563
	Mean Square Error (MSE)	0.000052	0.000021	0.000053	0.000055	0.000034	0.000035	0.000025	0.000071	0.000024	0.000022	0.000038	0.000021	0.000021
	RMSE	0.007226	0.004616	0.007253	0.007393	0.005832	0.005922	0.005031	0.008440	0.004896	0.004698	0.006124	0.004620	0.004545
	MAPE (%)	2.019	0.585	1.632	1.904	1.254	1.341	0.777	1.849	0.716	0.596	1.354	0.606	0.544
**ML4**	Mean Absolute Deviation (MAD)	0.0032556	0.0013282	0.0040002	0.0040240	0.0005095	0.0039669	0.0038250	0.0027744	0.0031787	0.0013349	0.0040280	0.0013341	0.0010268
	Mean Square Error (MSE)	0.0000180	0.0000020	0.0000171	0.0000180	0.0000004	0.0000160	0.0000231	0.0000096	0.0000147	0.0000020	0.0000166	0.0000020	0.0000012
	RMSE	0.0042461	0.0014021	0.0041331	0.0042417	0.0006236	0.0039962	0.0048019	0.0030969	0.0038287	0.0014056	0.0040709	0.0014080	0.0011007
	MAPE (%)	1.096	0.445	1.348	1.356	0.172	1.336	1.289	0.935	1.071	0.450	1.357	0.450	0.346

Source (Sharan et al., 2024b)

**Fig. 11:**
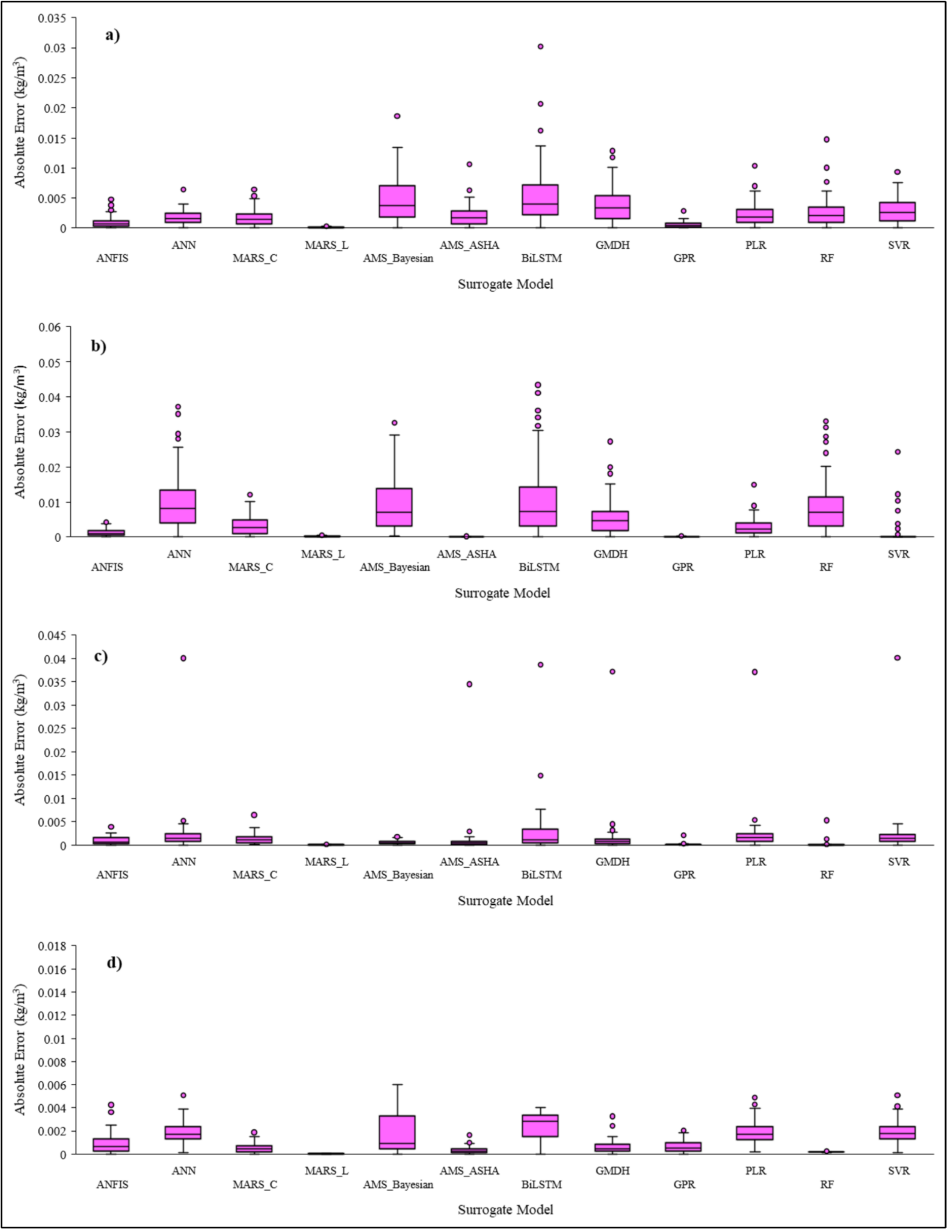
Boxplots of absolute errors between actual and predicted salt concentration during the training stage at** a** ML1, **b** ML2, **c** ML3, and **d** ML4 using all 12 surrogate models. Pink dots indicate outliers

The boxplots of absolute error between the observed and computed values for all MLs during the surrogate model training are given in Fig. 11. The absolute error close to zero indicates a better-performing model.

Different MLs had different surrogate models that outperformed amongst each other. Hence, to further narrow it down and choose the best performing models, Shannon’s entropy weights (EW) were used to rank the model (Sharan et al., 2024b). The EW method was applied to identify the best-performing surrogate model based on their assigned weights (Sharan et al., 2024b). A higher EW for a performance metric signifies a stronger influence on determining the weight of individual models. In other words, larger EW values indicate that the corresponding performance metric plays a more significant role in the decision-making process. The EWs for each prediction model were calculated to rank their effectiveness in predicting salt concentrations across four MLs. The ranking of the models was determined entirely by their EWs, as presented in Table 4.

**Table 4:** Entropy weight of the individual prediction models

Standalone models	ML1		ML2		ML3		ML4	
	EW	Rank	EW	Rank	EW	Rank	EW	Rank
ANFIS	0.745	7	0.605	9	0.425	9	0.191	7
ANN	0.918	1	0.436	11	0.915	1	0.598	2
MARS_C	0.697	8	0.994	1	0.423	10	0.151	10
MARS_L	0.572	9	0.820	5	0.300	12	0.125	11
AMS_Bayesian	0.912	2	0.639	8	0.444	7	0.806	1
AMS_ASHA	0.877	5	0.926	2	0.505	6	0.201	6
Bi-LSTM	0.533	11	0.315	12	0.542	5	0.093	12
GMDH	0.508	12	0.815	7	0.351	11	0.172	8
GPR	0.891	4	0.817	6	0.673	4	0.238	5
PLR	0.904	3	0.833	4	0.822	3	0.525	3
RF	0.549	10	0.446	10	0.442	8	0.152	9
SVR	0.816	6	0.865	3	0.840	2	0.341	4

Source (Sharan et al., 2024b) *EW* = Entropy Weight, *ANFIS* = Adaptive Neuro Fuzzy Inference System, *ANN* = Artificial Neural Network, *MARS_C* = Multivariate Adaptive Regression Spline with Piecewise Cubic Spline, *MARS_L* = Multivariate Adaptive Regression Spline with Piecewise Linear Spline, *AMS_Bayesian* = Bayesian Optimization tuned Automatic Model Selection, *AMS_ASHA* = Asynchronous Successive Halving Algorithm tuned Automatic Model Selection, *Bi-LSTM* = Bi-directional Long Short Term Memory Network, *GMDH* = Group Method of Data Handling, *GPR* = Gaussian Process Regression, *PLR* = Probabilistic Linear Regression, *SVR* = Support Vector Regression

**Fig. 12:**
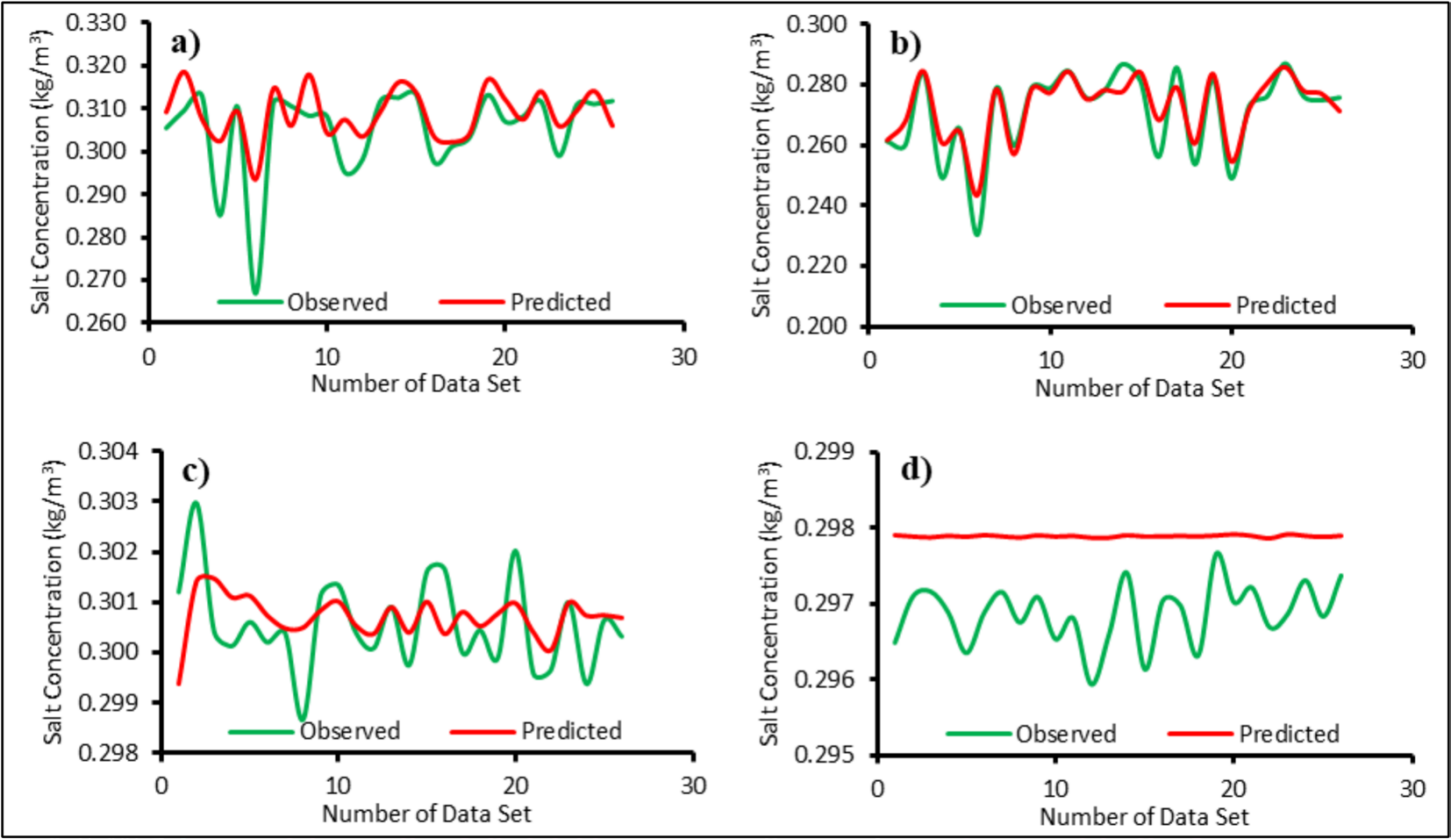
Observed versus predicted salt concentrations using surrogate ensemble models at **a** ML1, **b** ML2, **c** ML3, and **d** ML4

The top 5 ranked surrogate models (Table 4) were used to form ensemble surrogate models for each ML. The prediction results of surrogate ensemble models are shown in Fig. 12. The performance of each ensemble is also shown in Table 3. The ensemble outperforms standalone surrogates. The surrogate ensemble for ML4 (Fig. 12d) did not predict the variations as shown by other surrogate ensembles for MLs 1–3. However, the results of all surrogate ensemble models performed better than the standalone surrogate models.

### Digital twin

The four surrogate ensembles for each ML were linked with MOGA to develop a multi-objective saltwater intrusion management model (simulation-optimisation model; S–O) for the Tagabe coastal aquifer. The management model aimed to balance two conflicting objectives: maximising production well pumping while minimising barrier well pumping. The optimisation process was constrained by the requirement to maintain salt concentrations below a specified maximum (0.5 kg/m^3^) at various MLs. Multiple simulations were conducted using different combinations of parameters, with the optimal values applied to the optimisation framework. The results from the S–O model are used to make informed decisions in managing the freshwater pumping rates at Tagabe coastal aquifer.

The 3D numerical simulation model results, surrogate ensemble model results, and S–O model results were used as a DT for Tagabe coastal aquifer. The DT working framework is given in Fig. 3. The scenarios tested under DT are given in the “Digital Twin” section. The different illustrative field sensors triggered and fed the data to the S–O model were simulated.

**Fig. 13:**
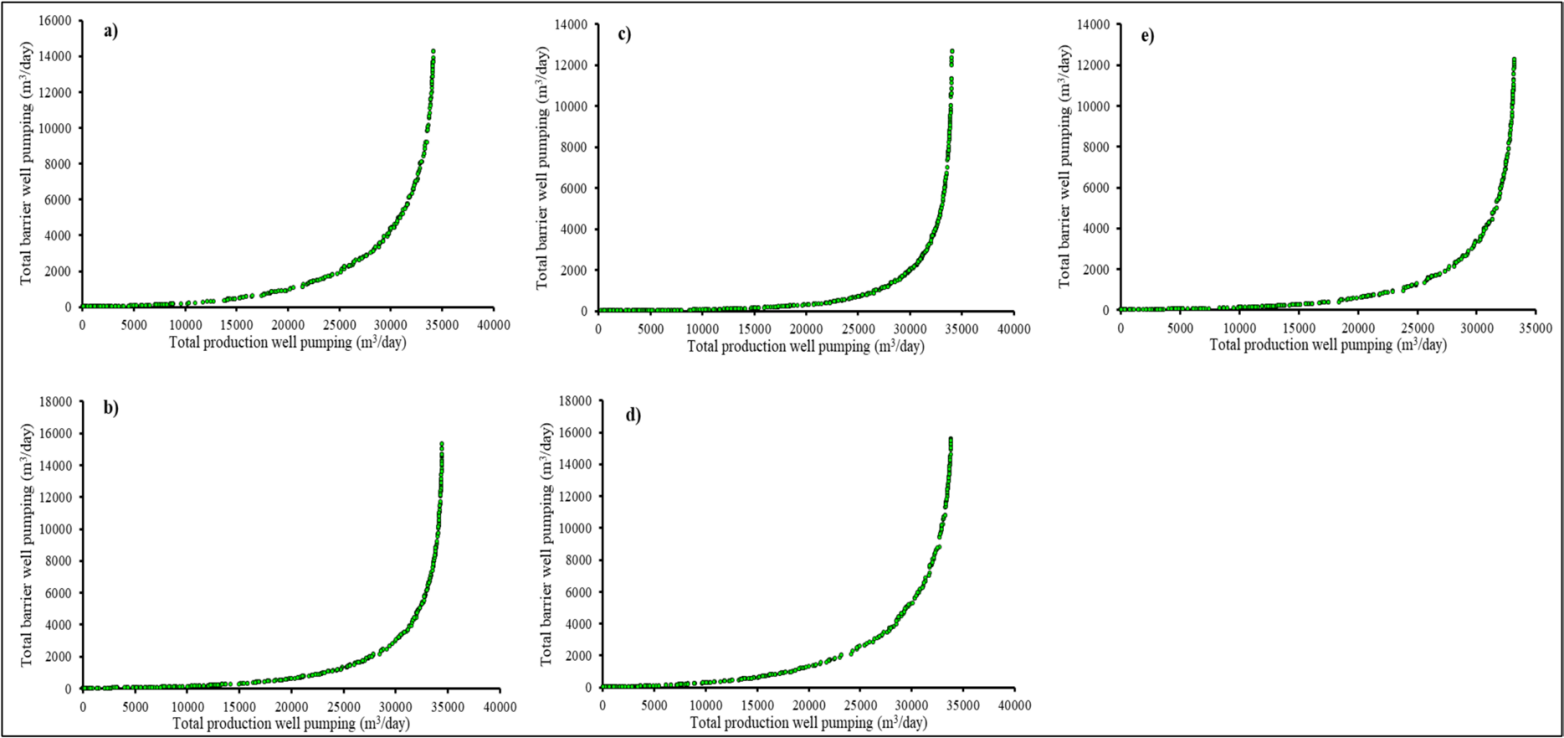
Pareto optimal fronts of the linked simulation-optimisation management model based on different scenarios for digital twin. **a** Scenario 1 with a maximum allowable salt concentration of 0.45 kg/m^3^. **b** Scenario 2 with a maximum allowable salt concentration of 0.55 kg/m^3^. **c** Scenario 3 with a maximum allowable salt concentration of 0.75 kg/m^3^. **d** Scenario 4 with a maximum allowable salt concentration of 0.90 kg/m^3^. **e** Scenario 5 with a maximum allowable salt concentration of 1.15 kg/m^3^

Initially, a S–O model was developed based on a maximum allowable salt concentration of 0.5 kg/m^3^ (Sharan et al. (2024b). For this study and scenario 1, the sensors triggered and sent the data indicating that the maximum salt concentration was decreased from 0.5 to 0.45 kg/m^3^. Hence, optimal pumping rates need to be simulated based on the new concentration data received by the DT. The population size of 2000 executed 16,000 generations to achieve the Pareto optimal solutions. The crossover and mutation rates were set at 0.92 and 0.1, respectively. The migration parameter was set to move in a forward direction with a fraction of 0.2 and an interval of 20. A population fraction of 0.7 was applied to the Pareto front, yielding 1400 non-dominated optimal solutions out of 2000 individuals (2000 × 0.7). Both the function and constraint tolerances were established at 1 × 10^–4^. The optimisation process required 7286 s to reach convergence and simulate the optimal solutions. The Pareto optimal generated for scenario 1 is given in Fig. 13a. The maximum pumping from all bores in Tagabe aquifer with a maximum salt concentration of 0.45 kg/m^3^ is predicted as 11,500 m^3^/d with 219 m^3^/d extraction from the barrier well. Increasing the pumping from production wells will also increase the pumping from barrier wells, as shown in Fig. 13a.

Similarly, the Pareto optimal fronts for scenarios 2–5 were generated using the same method and optimisation algorithm as used for scenario 1. However, the maximum allowable salt concentrations varied as triggered by the illustrative sensors from the field. The Pareto optimal fronts for scenarios 2–4 are shown in Fig. 13b–e, respectively. The optimisation time taken to generate the Pareto optimal fronts for scenarios 2–5 was 11,353 s, 16,532 s, 16,577 s, and 18,655 s, respectively. Increasing the allowable salt concentration took more time than the lower concentration constraint. The maximum production well pumping for scenarios 2–5 are 15,107 m^3^/d, 17,317 m^3^/d, 10,044 m^3^/d, and 15,155 m^3^/d, respectively. Similarly, based on these maximum production well pumping, the barrier well pumping for scenarios 2–5 are 280 m^3^/d, 202 m^3^/d, 293 m^3^/d, and 269 m^3^/d, respectively.

Roy and Datta (2017a, 2017b) and Lal and Datta (2018) used a similar approach to simulate the Pareto optimal fronts. However, they compared a small number of surrogate models (fewer than five) and created ensembles from them. In contrast, this study developed and compared a total of 12 surrogate models. The five top-performing models identified through Shannon’s entropy-based decision theory were then used to create a weighted-average ensemble model. Previous studies, such as those by Bhattacharjya and Datta (2005) and Hussain et al. (2015), applied a linked S–O technique but relied on single surrogate models combined with optimisation algorithms. By incorporating multiple surrogates into an ensemble, this study reduced the uncertainty in predicting salt concentrations. The ensembles developed here outperformed standalone surrogate models significantly.

### Validation of Pareto optimal fronts

The performance of the surrogate-based saltwater intrusion management model was evaluated by examining its ability to meet the predefined constraints. The results demonstrated that the saltwater concentrations predicted by the optimisation model, which utilised surrogate models, consistently remained below the maximum permissible levels at all monitoring locations. This indicates that the model successfully adhered to the constraints without any violations throughout the optimisation process. Furthermore, the saltwater concentrations were found to be close to the specified limits, suggesting that the optimisation model effectively converged near the upper bounds of the constraints.

The Pareto fronts generated by the surrogate ensemble–CEMOGA optimisation models at each ML were verified and validated. To achieve this validation, five randomly selected production and barrier well pumping solutions were added as inputs into the 3D numerical model. The resulting salt concentrations from the numerical model were then compared with the salt concentrations predicted by the S–O model. The outcomes of this validation process are presented in Table 5. The relative error (RE) between the observed and computed ranges between 0.5 and 9.99%. The RE is less than 10%, indicating that optimal solutions converged for the upper limit of the constraints.

**Table 5:** Validation of Pareto optimal front solutions with the numerical simulation model for all five scenarios of the digital twin approach

Scenario	Solution number	ML1			ML2			ML3			ML4		
		NSM (kg/m^3^)	ESM (kg/m^3^)	RE (%)	NSM (kg/m^3^)	ESM (kg/m^3^)	RE (%)	NSM (kg/m^3^)	ESM (kg/m^3^)	RE (%)	NSM (kg/m^3^)	ESM (kg/m^3^)	RE (%)
	1	0.297	0.320	7.858	0.296	0.283	4.340	0.296	0.295	0.479	0.296	0.325	9.710
	2	0.300	0.320	6.549	0.297	0.287	3.424	0.297	0.299	0.808	0.296	0.325	9.677
1	3	0.301	0.320	6.364	0.297	0.288	3.110	0.297	0.300	0.970	0.296	0.325	9.642
	4	0.301	0.320	6.289	0.297	0.288	2.968	0.297	0.300	0.921	0.297	0.325	9.637
	5	0.301	0.320	6.250	0.297	0.288	2.876	0.297	0.300	0.944	0.297	0.325	9.627
2	1	0.297	0.326	9.630	0.296	0.283	4.244	0.296	0.300	1.078	0.296	0.315	6.322
	2	0.297	0.326	9.630	0.296	0.290	2.181	0.296	0.300	1.185	0.296	0.310	4.635
	3	0.299	0.326	9.042	0.297	0.287	3.231	0.297	0.300	1.020	0.296	0.320	7.999
	4	0.300	0.326	8.661	0.297	0.289	2.652	0.297	0.300	0.986	0.296	0.320	7.981
	5	0.299	0.326	8.893	0.297	0.288	3.015	0.297	0.300	1.023	0.296	0.320	8.010
3	1	0.301	0.330	9.737	0.297	0.287	3.415	0.297	0.300	1.051	0.296	0.326	9.958
	2	0.301	0.330	9.715	0.297	0.282	5.026	0.297	0.300	1.038	0.296	0.326	9.842
	3	0.300	0.330	9.820	0.297	0.289	2.839	0.297	0.300	1.076	0.296	0.325	9.736
	4	0.297	0.325	9.393	0.296	0.289	2.222	0.296	0.300	1.313	0.296	0.325	9.702
	5	0.300	0.325	8.467	0.297	0.289	2.497	0.297	0.300	1.147	0.296	0.325	9.691
4	1	0.301	0.326	8.463	0.297	0.284	4.442	0.297	0.300	1.010	0.296	0.325	9.750
	2	0.299	0.325	8.756	0.297	0.281	5.207	0.297	0.300	0.996	0.296	0.325	9.779
	3	0.300	0.326	8.748	0.297	0.288	3.002	0.297	0.300	1.037	0.296	0.325	9.768
	4	0.297	0.324	9.223	0.296	0.287	3.215	0.296	0.300	1.208	0.296	0.325	9.797
	5	0.297	0.327	9.954	0.296	0.290	2.177	0.296	0.300	1.183	0.296	0.325	9.791
5	1	0.300	0.321	7.114	0.297	0.285	3.820	0.297	0.300	1.086	0.296	0.325	9.697
	2	0.297	0.322	8.331	0.296	0.282	4.754	0.296	0.300	1.177	0.296	0.325	9.700
	3	0.300	0.324	8.081	0.297	0.288	2.942	0.296	0.300	1.169	0.296	0.325	9.697
	4	0.297	0.326	9.897	0.297	0.284	4.470	0.296	0.300	1.150	0.296	0.325	9.678
	5	0.297	0.325	9.282	0.296	0.286	3.314	0.297	0.300	1.012	0.296	0.325	9.708

*NSM* – Numerical simulation model; *ESM* = ensemble surrogate model; *RE* = absolute relative error between the simulated and the predicted salt concentrations; *ML* = Monitoring location; *C* = Concentration

The results of this study align with those reported by Roy and Datta (2017b), who indicated that relative errors below 10% are suitable for devising an optimal management strategy using an integrated S–O approach. Therefore, it can be concluded that the machine learning-based surrogate ensemble model developed in this study could serve as an effective substitute for the complex nonlinear, density-dependent SEAWAT model in optimising groundwater management for the Tagabe coastal aquifer. Furthermore, this methodology could potentially be applied to other aquifers as well.

The current study used the methodology developed by Sharan et al. (2024b). Hence, some of the results overlap. The Limitation of this study was the small dataset used to train the surrogate models with 130 input–output patterns. Hence, deep learning models did not perform well during the training and testing stages. Moreover, the illustrative sensors were used during the development of the digital twin for Tagabe coastal aquifer instead of real sensors. Getting government approvals from Vanuatu, the cost of buying the sensors, smart water quality metres, solar panels, and other types of equipment needed to feed data from the field to the virtual space (virtual Tagabe aquifer) was not available. Hence, the digital twin was developed using illustrative data. In the future, with funding, the illustrative sensors and scenarios will be replaced with actual sensors and field data.

## Summary and conclusions

Saltwater intrusion (SWI) is a major environmental concern for coastal aquifers in Pacific Island countries, including Port Vila and Vanuatu. Based on current pumping regimes, sea level rise, and anthropogenic activities, Tagabe coastal aquifer would have SWI, as demonstrated by the 3D numerical model simulation in this study. To manage SWI in Tagabe coastal aquifer, optimal pumping patterns were generated using machine learning-based surrogate ensemble models and a simulation-optimisation management model (S–O). The S–O model was later used to create the digital twin (DT) of Tagabe coastal aquifer.

The current study developed a digital twin (DT) framework for Tagabe coastal aquifer. The DT was developed using a 3D numerical simulation model, surrogate and ensemble models, and S–O. The DT framework was designed and linked using the illustrative field data. Due to funding constraints and government approvals, the real field sensors were not placed in Tagabe coastal aquifer. Hence, illustrative sensors were used to generate five different scenarios. These illustrative sensors triggered and sent the data from the physical space (Tagabe aquifer) to the virtual space with changes in the salt concentration values at different MLs. Based on these illustrative data, the maximum allowable salt concentrations for scenarios 1 to 5 were 0.45, 0.55, 0.75, 0.90, and 1.15 kg/m3, respectively. Using these allowable salt concentrations and maximum and minimum pumping values, the simulation model (surrogate ensemble) was optimised using Controlled Elitist Multiple Objective Genetic Algorithm.

The S–O gave Pareto optimal fronts or solutions that could be used to maximise the pumping from the production wells and minimise the pumping from the barrier wells. Five sets of Pareto optimal fronts were generated for each constraint (different salt concentrations). Scenario 3 (concentration of 0.75 kg/m3) gave higher pumping (17,317 m^3^/d) from the production wells and lowest pumping (202 m^3^/d) from the barrier wells, compared with other scenarios. However, the selected compromise solutions or the Pareto frontier (knee of the curve) of generated optimal solutions kept on increasing as the maximum allowable salt concentrations increased. Hence, scenario 5 had the higher Pareto frontier or solutions of 31,676 and 5000 m^3^/d pumping from production wells and barrier wells, respectively. The results from the S–O model were validated using randomly selected solutions from each scenario. The relative error was less than 10% when outputs from the numerical model were compared with the outputs from the S–O model.

To the author’s best knowledge, the application of DT in managing SWI has not been applied before. The results from the study indicated that DT works for a coastal aquifer in managing the SWI. The methodology developed and implemented in this study is of global significance and could be used to manage water resources wisely. The study demonstrated that the DT is vital in predicting future scenarios, changes in pumping patterns, and other uncertainties. DT helped attain outcomes based on feedback information from continuous monitoring with sensors with a smaller time interval (a month in this study). Overall, the study demonstrated that DT helps modify management strategies more efficiently and effectively based on field data transmitted back.

Using the DT of an aquifer offers a powerful tool for enhancing water resource management and sustainability. By creating a virtual replica of the aquifer, this technology allows for real-time monitoring, simulation, and analysis of the aquifer’s behaviour under various conditions. This helps in understanding complex hydrological processes, predicting the impacts of human activities and climate change, and optimising water extraction and conservation strategies as demonstrated by the results in this study. The DT can integrate data from sensors, historical records, and predictive models to provide actionable insights, leading to more informed decision-making and improved management of vital water resources.

The use of field smart sensors, smart water quality metres, smart information boards, and other components of DT would be considered for future scope of work in Tagabe coastal aquifer, including other Pacific Island countries. Moving forward, the implementation of a fully operational DT system for the Tagabe coastal aquifer will require installation of smart sensors capable of continuously monitoring salinity, groundwater levels, and other key hydrogeological parameters. Integration of these sensor networks with the DT platform will enable real-time data assimilation and adaptive model updating, improving prediction accuracy and management responsiveness. Key challenges anticipated include ensuring sensor reliability and maintenance in a coastal environment, establishing robust data communication infrastructure, addressing data gaps or noise, and managing the computational resources necessary for real-time simulation. Overcoming these challenges will be critical to realising the full potential of DT technology for effective coastal groundwater management.

## Data Availability

No datasets were generated or analysed during the current study.
